# A Joint Extraction Model for Entity Relationships Based on Span and Cascaded Dual Decoding

**DOI:** 10.3390/e25081217

**Published:** 2023-08-16

**Authors:** Tao Liao, Haojie Sun, Shunxiang Zhang

**Affiliations:** College of Computer Science and Engineering, Anhui University of Science and Technology, Huainan 232001, China

**Keywords:** entity relation extraction, span, decode, cascade, neural network

## Abstract

The entity–relationship joint extraction model plays a significant role in entity relationship extraction. The existing entity–relationship joint extraction model cannot effectively identify entity–relationship triples in overlapping relationships. This paper proposes a new joint entity–relationship extraction model based on the span and a cascaded dual decoding. The model includes a Bidirectional Encoder Representations from Transformers (BERT) encoding layer, a relational decoding layer, and an entity decoding layer. The model first converts the text input into the BERT pretrained language model into word vectors. Then, it divides the word vectors based on the span to form a span sequence and decodes the relationship between the span sequence to obtain the relationship type in the span sequence. Finally, the entity decoding layer fuses the span sequences and the relationship type obtained by relation decoding and uses a bi-directional long short-term memory (Bi-LSTM) neural network to obtain the head entity and tail entity in the span sequence. Using the combination of span division and cascaded double decoding, the overlapping relations existing in the text can be effectively identified. Experiments show that compared with other baseline models, the F1 value of the model is effectively improved on the NYT dataset and WebNLG dataset.

## 1. Introduction

Entity–relationship extraction means to extract entity relationships from unstructured text [1,2] and convert them into structured data by analyzing unstructured text. Entity–relationship extraction is very important for building knowledge graphs and question-answering systems [3], and information retrieval tasks [4,5] play a crucial role [6]. The entity–relationship triple is one of the basic representation methods of entity relationships. A triple in the form of <head entity, relation, tail entity> consists of two entities and the relationship between them, which represents the semantic relationship between entities in the text.

The two primary kinds of existing entity–relationship extraction methods are the pipeline extraction method and the joint model extraction method [7]. Among them, the pipeline extraction method divides the relationship extraction task into two independent subtasks, firstly, identifying the entities in the given text, and then identifying the relationship between entities [8,9,10]. The joint model extraction method recognizes the entities and the relationship between entities at the same time. Although the above two methods have achieved good results, the existing models still face problems such as entity nesting, overlapping relationships, and data noise. In the overlapping relationship problem, the overlapping relationship includes single entity overlap (SEO) and entity pair overlap (EPO) [11]. In this paper, this category is also used for the division, as shown in Table 1. In the sentence ‘The city of Aarhus, whose mayor is Jacob Bundsgaard, is served by Aarhus Airport’, there are two entity–relationship triples ‘< Aarhus, leaderName, Bundsgaard >’ and ‘< Aarhus airport, cityServed, Aarhus >’. There is a ‘leaderName’ relationship between the entity ‘Aarhus’ and the entity ‘Bundsgaard’, at the same time, and there is a ‘cityServed’ relationship between the entity ‘Aarhus’ and the entity ‘Aarhus airport’. We call this type of overlapping relationship as single entity overlap. In the sentence ‘News of the list’s existence unnerved officials in Khartoum, Sudan ’s capital’, there are two entity–relationship triples ‘< Sudan, capital, Khartoum > and < Sudan, contains, Khartoum >’. There are two kinds of relationships between entity ‘Sudan’ and entity ‘Khartoum’, which are the ‘capital’ relationship and the ‘contains’ relationship. We call this type of overlapping relationship as entity pair overlap.

In this paper, we propose a novel joint entity–relationship extraction model based on a span and cascaded dual-decoding method (SCDM) to solve the problem of overlapping relationship. First, we use the Bidirectional Encoder Representations from Transformers (BERT) pretrained language model to process the text, convert them into the tokens, and then use the random span division mechanism to divide the tokens to form span sequences. Second, we perform relation decoding on span sequences in combination with a predefined set of relation types to obtain the relation types that exist between span sequence entities. Third, integrating the relationship type obtained by the relationship decoding layer for entity decoding, in the entity decoding part, we first obtain the head entity and then obtain the tail entity. The head entity is decoded by fusing the relation type feature, and the tail entity is obtained by fusing the relation type feature and the head entity. Through the above steps, the model proposed can effectively solve the overlapping relationship, and use the dual-decoding mechanism, which can improve the training speed and reduce the error transmission.

Overall, the significant contributions of this paper are as follows:

- A novel joint entity–relationship extraction model, SCDM, based on the span and a cascaded dual decoding are proposed. Different from existing methods, this paper performs the span division after the BERT preprocessing to form span sequences, and subsequent decoding tasks are performed within the span sequences. The issue of overlapping relations in the text is successfully resolved.
- This method uses a cascaded decoding mechanism, which has the advantages of a fast learning speed and a reduced error transmission. First, it decodes the relationship between entities in the span sequences, and then it decodes the entities in the span sequences to obtain the head entity and the tail entity. The relationship type information obtained by the relation decoding layer is fused when decoding the head entity, and the relationship type information and the acquired head entity information are fused when decoding the tail entity.
- Through the analysis of the NYT dataset and the WebNLG dataset, when compared to the current models, the methodology suggested in this research produced the best results.

## 2. Related Work

An essential task in the field of natural language processing (NLP) is extracting entity connections from unstructured text, which is also a necessary step in building a knowledge graph to provide support for downstream related tasks [12,13]. At present, the mainstream entity–relationship extraction methods can be roughly divided into two types: pipeline entity–relationship extraction methods and joint entity–relationship extraction methods.

### 2.1. The Pipeline Extraction Model

The relationship extraction task is divided into two separate subtasks using the pipeline extraction approach. Nayak T. et al. [14] introduced the use of the Bi-LSTM neural network model and attention mechanism to obtain long-distance dependent information, effectively solving the problem of facing sentences that are longer and have long distances between entities in the sentence. Zeng D. et al. [15] introduced the use of deep convolutional neural networks to extract vocabulary and sentence-level features in the text, which effectively solved the problem of classifying the relationship between entity pairs that required complex preprocessing. Guo X. et al. [16] introduced the use of a neural network combining recurrent neural network (RNN) and convolutional neural network (CNN) and adding an attention mechanism to complete the relationship classification, extract higher-level text information and obtain the feature information of sentences. Guo Z. et al. [17] introduced adding entity type and relationship alias information and inputting them into the graph convolutional neural network to improve the effect of the entity–relationship extraction.

### 2.2. The Joint Extraction Model

The pipeline method does not need to manually construct features and has high accuracy, so it is widely used, but because the error in the entity recognition task may be transmitted to the relationship classification task, it causes the problem of error propagation [1]. Therefore, in recent years, researchers have gradually focused on the research of joint entity–relationship extraction methods. Zeng X. et al. [11] introduced an end-to-end model based on the copy mechanism. That model used the copy mechanism to solve the problem of overlapping relationships and achieved excellent results. Copying text information is likely to cause information redundancy and relatively complex calculations. Luo L.et al. [18] introduced a tagging scheme for solving overlapping problems and proposed an Att-Bi-LSTM-CRF model to solve the problem of entity–relationship extraction in the biological domain. Ma Y. et al. [19] introduced a self-training entity–relationship extraction model in document-level text, which mainly solved the problems of high memory consumption and manual annotation in document-level entity-relationship extraction. Bhartiya A. et al. [20] first analyzed the problems existing in the original dataset and introduced a dataset, DiS-ReX, for the entity-relationship extraction, and then provided a benchmark. Hwang W.et al. [21] introduced an end-to-end extraction system applied to the legal field, which could bring convenience to lawyers’ statistics and analysis of legal data. Xie Y. et al. [22] improved the performance of the entity-relationship extraction model by enhancing evidence in document-level entity-relationship extraction.

In recent years, Shang Y. et al. [23] introduced a method using one step and one module to extract entity and relations. They showed an annotation strategy and a decoding strategy to solve the overlapping relations. Ye D. et al. [24] introduced a new method to obtain the representation of the relationship between span sequences. The packaging strategy oriented by similar spans effectively solved the problem of overlapping relationships in the text. Wei Z. et al. [25] introduced a cascaded binary tagging framework for entity-relationship extraction and analyzed the framework theoretically. It not only significantly improved the effect in the case of BERT pretraining but also significantly improved the extraction effect even without pretraining. Ma L. et al. [26] conducted research on the basis of Wei Z. et al. [25], which introduced the use of cascaded double-decoding methods for entity–relationship extraction. First, the relationships existing in the text were decoded, and then the head and tail entities in the text were decoded. Although this method achieved good results, it only partially solved the problem of overlapping relationships.

This paper is also aimed at the problem of overlapping relationships in the text. Different from Ma L. et al. [26], the method proposed in this paper uses random spans to form span sequences after the BERT encoding layer. A span partitioning of the tokens can divide entities that may have overlapping relationships into the same span sequence to solve the overlapping relationship problem. The length of the span sequence is effectively set to take advantage of the long-distance relationship between entities in the text.

## 3. Methodology

### 3.1. Overview Network Architecture

**Figure 1:**
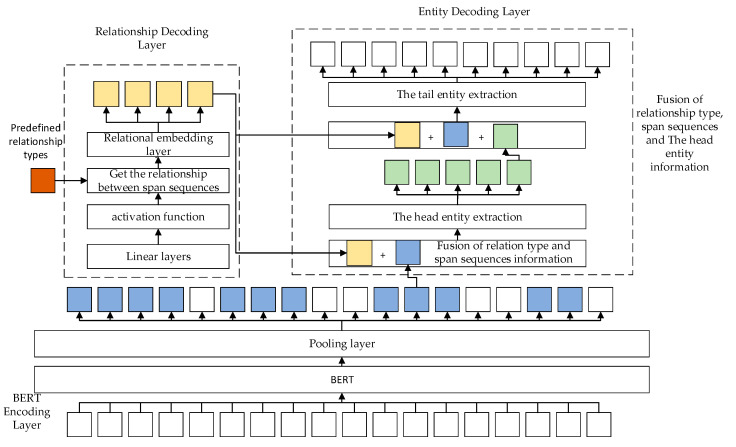
The framework of the model.

This section describes the joint entity–relationship extraction model proposed in this paper based on the span and a cascaded decoding. Compared with the original joint model of entity–relationship extraction, if there are overlapping relationships in the text, the effect of the entity–relationship extraction will not be ideal. On the whole, the model is divided into three layers, a BERT decoding layer, a relationship decoding layer, and an entity decoding layer. The entity decoding layer includes two parts: the head entity extraction and the tail entity extraction. In this model, we first input the text into the BERT encoding layer, use the BERT-BASE-CASED pretrained language model to convert the text into vectors, and randomly divide the vectors to form span sequences. Then, we use linear layers to get the type of relationship between entities within the span sequences. Finally, the span sequences information is combined with the relationship type feature to extract the head entity and tail entity in the span sequence. The model is shown in Figure 1.

Where blue represents the span sequences, and the number of blue-colored blocks represents the size of the span sequence; yellow represents the relation type obtained by the relationship decoding layer; orange represents the predefined relation type, which contains 24 relation types in the NYT dataset, and the WebNLG dataset contains 246 relationship types; green indicates the head entity obtained by the entity decoding layer. In this model, we use the BERT encoder to generate tokens and divide them into span sequences; we use the relation decoder to get the relations in the span sequence; and we use the entity decoder to get the head and tail entities.

### 3.2. BERT Encoder Layer

We use the BERT pretrained language model to encode texts to capture semantic information between texts. The main task of the encoding layer is to encode the text information into matrix vectors. The encoding layer uses the BERT pretrained language model to obtain the semantic features of the sentence. The semantic features of the sentence are expressed as $X=[x_{1},x_{2},x_{3},x_{4}\ldots x_{n}]$, which contains the prior knowledge obtained by BERT (BERT-BASE-CASED) in the pretraining stage.

Firstly, the input text sequence is represented as word vectors through the embedding layer, and the *i*-th token in the processed vectors is represented as formula (1):

(1) $$e_{i}=W_{token}\left(t_{i}\right)$$

where $W_{token}\left(t_{i}\right)$ is represented as a token embedding. Then, we input it into the BERT (BERT-BASE-CASED) pretraining model for encoding. The BERT pretraining model contains 12 hidden layers, and the size of each hidden layer is 768. The encoded result in the sentence is shown in formula (2):

(2) $$H_{b}=BERT(E)$$

where E represents the sequence of tokens formed after token embedding, and the semantic representation $H_{b}$ of the sentence is obtained from the text through the BERT model, and it is input into the pooling layer to generate the input of the relational decoding layer, as shown in formula (3):

(3) $$H_{b}^{\prime }=Pooling(H_{b})$$

### 3.3. Relationship Decoding Layer

Since there are many kinds of relationships between entities in the span sequences, decoding the relationship type in the text is the main task of this layer. Determining the relationships that exist in span sequences is similar to a multilabel classification problem [26]. Span division is performed on the vectors generated by the BERT decoding layer to form a span sequence, as shown in formula (4):

(4) $$S^{i}=\left(s_{i},s_{i+1},\ldots ,s_{i+n}\right)$$

where (i, i + 1,⋯, i + *n*) represents the size of the span sequence formed after the division. The relationship decoding layer decodes the relationship type between entity pairs within span sequences. Given a predefined set of relationship types, as shown in formula (5):

(5) $$R^{t}=\left(r_{1},r_{2},\ldots ,r_{n}\right)$$

where *n* is the size of the relation type $R^{t}$, first, we input the span sequences into the linear layers to obtain the information in the span sequences, and then we use the sigmoid activation function to calculate the probability of the relationship type between entities, as shown in formula (6):

(6) $$P^{r}=\varphi \left(W^{r}h+b\right)$$

We set the relationship filtering threshold. If the value of $P^{r}$ is greater than the value of the relationship filtering threshold, it is considered to be a valid relationship type, otherwise, it is considered to be an invalid relationship type. We embed the relationship type in the span sequences into the vector for the subsequent entity decoding layer, as shown in formula (7):

(7) $$V^{r}=\left(v_{1},\;v_{2},\ldots ,v_{n}\right)$$

### 3.4. Entity Decoding Layer

#### 3.4.1. Head Entity Extraction

The main task of the head entity extraction is to extract head entities in relation types, which are obtained from the relation decoding layer. First, the span sequences and relation type information are fused; then, they are input into the Bi-LSTM neural network to obtain the information in the span sequences, where the output representation of the hidden layer of the Bi-LSTM neural network is shown in formula (8):

(8) $$H^{h}=Bi-LSTM\left(S^{i};V^{r}\right)$$

The probability of the head entity in the relation type to which the entity belongs in the span sequences is determined using the softmax activation function. As shown in formulas (9) and (10):

(9) $$P_{head}^{start}=Softmax\left(W_{h}^{s}*X+b_{h}^{s}\right)$$

(10) $$P_{head}^{tail}=Softmax\left(W_{h}^{t}*X+b_{h}^{t}\right)$$

where $W_{h}^{s}$ represents the weight matrix for obtaining the starting position, $W_{h}^{t}$ represents the weight matrix for obtaining the end position, X represents the matrix formed by fusing the final span sequences and relationship type, and $b_{h}^{s}$ and $b_{h}^{t}$ represent obtaining different position offsets.

#### 3.4.2. Tail Entity Extraction

The main task of the tail entity extraction is to extract tail entities in relation types. First, the span sequences, head entity information, and relationship type information are fused; then they are input into the Bi-LSTM neural network to obtain the information in the span sequences, where the output representation of the hidden layer of the Bi-LSTM neural network is shown in formula (11):

(11) $$H^{h}=Bi-LSTM\left(S^{i};V^{r};E^{h}\right)$$

where $E^{h}$ represents the head entity obtained by the head-entity extraction process. The probability of the tail entity in the relation type to which the entity belongs in the span sequences is determined using the softmax activation function, as shown in formulas (12) and (13):

(12) $$P_{tail}^{start}=Softmax\left(W_{t}^{s}*X+b_{t}^{s}\right)$$

(13) $$P_{tail}^{tail}=Softmax\left(W_{t}^{t}*X+b_{t}^{t}\right)$$

where $W_{t}^{s}$ represents the weight matrix for obtaining the starting position, $W_{t}^{t}$ represents the weight matrix for obtaining the end position, X represents the matrix formed by fusing the final span sequences and relationship type, and $b_{t}^{s}$ and $b_{t}^{t}$ represent obtaining different positions offset.

### 3.5. Loss Function

Relationship categorization and entity recognition are both multiclassification issues, so the cross-entropy loss function is used in the SCDM model. The relationship decoding layer, the head entity extraction, and the tail entity extraction’s loss functions are added to create the overall model’s loss function.

(14) $$L=c_{1}l^{r}+c_{2}l^{h}+c_{3}l^{t}$$

where $l^{r}$ represents the loss function of the relational decoding layer, $l^{h}$ represents the loss function of extracting the head entity in the entity decoding layer, and $l^{t}$ represents the loss function of extracting the tail entity in the entity decoding layer. $c_{1}$ represents the influence of the relational decoding layer in our model, and $c_{2}$ and $c_{3}$, respectively, represent the influence of the head entity extraction and tail entity extraction in the entity decoding layer in our model. The loss values of the relational decoding layer and the entity decoding layer are averaged to calculate the total loss of the model.

## 4. Experiments

### 4.1. Datasets

We evaluated the method proposed in this paper on two datasets, the NYT dataset [26] and the WebNLG dataset [27,28]. The training set in the NYT dataset has a total of 56,214 sentences, and the test set has a total of 5000 data and a total of 24 relationship types. The training set in the WebNLG dataset has a total of 5017 sentences, and the test set has a total of 703 data and a total of 246 relationship types. The division of the dataset is shown in Table 2.

### 4.2. Experimental Environment

The model proposed in this paper used the PyTorch framework and NVIDIA GeForce RTX 3090 (24 G) graphic processing unit (GPU), and its hardware and software are shown in Table 3.

### 4.3. Experimental Parameters

The experiment in this paper used the BERT-BASE-CASED pretrained language model. The word embeddings had a dimension of 300 and were randomly initialized. The location embedding dimension was 20. The training parameter settings are shown in Table 4.

### 4.4. Evaluation Methods

This paper used two methods to evaluate model performance: partial match and extract match. The partial match method [29] states that an extracted triple (h, r, t) is only considered right if its relation and the last letters in the names of the head entity and tail entity are accurate. A predicted triple is only considered right if its relation and the entire names of its head and tail entities are both correct, according to the exact match method [30,31]. Take note that only the final word of an entity name was annotated in both the training and test sets.

The evaluation indicators used in this experiment were precision, recall, and F1-score. The latter is the most important evaluation parameter in entity–relationship extraction.

### 4.5. Comparative Experiments

A comparison between the model in this research and the model from more recent years was conducted. Here are the comparative models:

1. CopyRe [19] proposed a method based on the replication mechanism and used one combined decoder or several independent decoders as two alternative approaches to the decoding process.
2. GraphRel [32] proposed a method using graph neural networks (GCN) and a copy mechanism to extract entity relation. It used GCN to make better use of spatial information and improve the extraction effect of models.
3. CopyRL [33] proposed a sequence-to-sequence model based on a copy mechanism and took into account the order of relationships; the model was able to provide superior outcomes thanks to the use of reinforcement learning.
4. CopyMTL [34] proposed a multitask learning method based on the replication mechanism, which enhanced the robustness of the model and could predict multitoken entities.
5. WDec [30] proposed a relation strategy for representation and a decoding method based on a pointer network to realize entity relation extraction.
6. AttentionRE [31] developed a supervised multihead self-attention technique to learn the token-level correlation for each individual connection type.
7. CasRel [25] proposed a binary cascaded tagging framework to eliminate the issue of text’s overlapping relations, and a related theoretical analysis was carried out.
8. DualDec [26] proposed a dual-decoding mechanism to extract entity–relationship triple, which first decoded the relationship in the text, and then decoded the entity pairs of a specific relationship.

The comparison between the model used in this paper and the baseline model on the NYT (N) dataset and the WebNLG (W) dataset yielded the findings shown in Table 5. In the table, we conducted comparative experiments on two datasets (NYT dataset and WebNLG dataset), taking the average of multiple experiments to obtain the data values. We strove to ensure the accuracy of the results. The data showed that the model proposed in this paper had improved accuracy, recall, and F1 score in the two datasets. In the N dataset, the F1 score of the partial match evaluation method was increased by 0.7%, and the F1 score of the exact match evaluation method was increased by 0.8%. In the W dataset, the F1 score of the partial match evaluation method was increased by 1%, and the F1 score of the exact match evaluation method was increased by 0.2%. This improvement was due to the use of the span to divide the text, so that the entity pairs that may have overlapping relationships were divided into the same span sequence, which effectively avoided the omission of the relationships between entity pairs, thereby improving the performance of the entity–relationship extraction.

### 4.6. Experimental Results on Different Relationships

**Figure 2:**
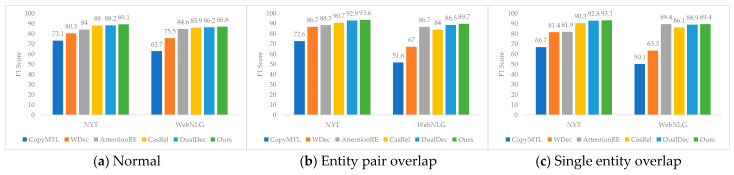
Results for various phrase kinds based on how closely they overlap when using the exact match method.

To investigate how the model in this research affected various relationship types, we also conducted comparative experiments. Since there were three relationship types in the text, normal, entity pair overlap and single entity overlap, comparative experiments were conducted on different relationship types to test the precision, recall, and F1 score, respectively, as shown in Figure 2. An entity–relationship triple ‘<h, r, t>’ was only considered right if its relation and the last letters in the names of the head entity and tail entity were accurate.

Our model was compared with existing models in the N dataset and the W dataset, which were CopyMTL [34], WDec [30] AttentionRE [31], CasRel [25], and DualDec [26]. From Figure 2, it can be seen that the model in this paper achieved the best results.

As shown in Figure 2, in the normal relationship, the F1 score of the model proposed in this paper was 0.9% and 0.6% higher than that of the DualDec model on the NYT dataset and WebNLG dataset. In the EPO relationship, the proposed model’s performance improved by 0.7% and 1.2% in the F1 score over the DualDec model on the NYT dataset and the WebNLG dataset, respectively. In the SEO relationship, the proposed model’s performance improved by 0.3% and 0.5% in the F1 score over the DualDec model on the NYT dataset and the WebNLG dataset, respectively.

## 5. Conclusions

According to the analysis of existing entity–relationship extraction methods, this paper proposed a joint entity–relationship extraction model based on the span and a cascaded dual decoding. The model first divided the word vectors based on the span after the BERT pretraining model to form new span sequences and then decoded the relationship type between entity pairs in the span sequence. Finally, the span sequences information was combined with the relationship type feature, and the head entity and tail entity in the span sequences were extracted using the Bi-LSTM neural network. Experimental results showed that the model achieved certain results. The next step will be to study the span sequences formed after the division and to study the relationship between entities in the span sequence and the possible relationship between span sequences, so as to achieve better extraction results when the text contains complex overlapping relationships.

## Figures and Tables

**Table 1:** The examples of normal, single entity overlap, and entity pair overlap. Single entity overlap means that two or more entities have a relationship with a certain entity; entity pair overlap means that there is more than one relationship between two entity pairs.

Type	Example Sentences and Entity Relationships	Graphic Representation
Normal	York is located in the England.	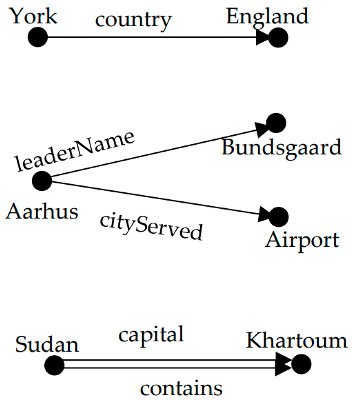
	{<York, country, England>}	
Single entity overlap	The city of Aarhus, whose mayor is Jacob Bundsgaard, is served by Aarhus Airport.	
	{<Aarhus, leaderName, Bundsgaard >, <Aarhus airport, cityServed, Aarhus>}	
Entity pair overlap	News of the list’s existence unnerved officials in Khartoum, Sudan’s capital.	
	{<Sudan, capital, Khartoum >, <Sudan, contains, Khartoum>}	

**Table 2:** Size of datasets.

Datasets	NYT		WebNLG	
	Training Set	Test Set	Training Set	Test Set
Normal	37,015	3264	1599	246
EPO	9781	979	224	26
SEO	14,737	1295	3407	457
Total	56,214	5000	5017	703
Relation type	24		246	

**Table 3:** Software and hardware environment.

Name	Environment
System	Windows
GPU	NVIDIA GeForce RTX 3090 (24 G)
Memory	56 G
Hard disk	2 T
Python version	Python 3.8
PyTorch version	1.8.0

**Table 4:** Training parameter settings.

Parameter	Value
BERT model	BERT-BASE-CASED
Learning rate	2e^−5^
Batch size	16
Epochs	100
Dropout	0.4
Optim	Adam
Position embedding size	20
Max span	10

**Table 5:** Compared with the baseline model. Experiments were carried out on the NYT (N) dataset and the WebNLG (W) dataset, respectively, on the partial match and exact match evaluation methods.

Methods	Partial Match						Exact Match					
	NYT(N)			WebNLG(W)			NYT(N)			WebNLG(W)		
	Pre.	Rec.	F1	Pre.	Rec.	F1	Pre.	Rec.	F1	Pre.	Rec.	F1
CopyRe	61.0	56.6	58.7	37.7	36.4	37.1	-	-	-	-	-	-
GraphRel	63.9	60.0	61.9	44.7	41.1	42.9	-	-	-	-	-	-
CopyRL	77.9	67.2	72.1	63.3	59.9	61.6	-	-	-	-	-	-
CopyMTL	-	-	-	-	-	-	75.7	68.7	72.0	58.0	54.9	56.4
WDec	-	-	-	-	-	-	88.1	76.1	81.7	88.6	51.3	65.0
AttentionRE	-	-	-	-	-	-	88.1	78.5	83.0	89.5	86.0	87.7
CasRel	89.7	89.5	89.6	93.4	90.1	91.8	89.1	89.4	89.2	87.7	85.0	86.3
DualDec	90.2	90.9	90.5	90.3	91.5	90.9	89.9	90.3	90.1	88.0	88.9	88.4
Ours	89.8	92.7	91.2	91.6	92.2	91.9	91.0	90.8	90.9	88.9	88.4	88.6

## Data Availability

Not applicable.
